# Establishment of a Prognostic Model for Hepatocellular Carcinoma Based on Endoplasmic Reticulum Stress-Related Gene Analysis

**DOI:** 10.3389/fonc.2021.641487

**Published:** 2021-05-21

**Authors:** Peng Liu, Jinhong Wei, Feiyu Mao, Zechang Xin, Heng Duan, Yan Du, Xiaodong Wang, Zhennan Li, Jianjun Qian, Jie Yao

**Affiliations:** ^1^ Medical College of Yangzhou University, Yangzhou, China; ^2^ School of Basic Medical Sciences, Southwest Medical University, Luzhou, China; ^3^ Department of General Surgery, First Affiliated Hospital of Dalian Medical University, Dalian, China; ^4^ Department of Hepatobiliary and Pancreatic Surgery, Northern Jiangsu People’s Hospital, Yangzhou, China

**Keywords:** hepatocellular carcinoma, The Cancer Genome Atlas, endoplasmic reticulum stress, signature, prognosis, multi-omics

## Abstract

Hepatocellular carcinoma (HCC) is one of the most common types of cancer worldwide and its incidence continues to increase year by year. Endoplasmic reticulum stress (ERS) caused by protein misfolding within the secretory pathway in cells and has an extensive and deep impact on cancer cell progression and survival. Growing evidence suggests that the genes related to ERS are closely associated with the occurrence and progression of HCC. This study aimed to identify an ERS-related signature for the prospective evaluation of prognosis in HCC patients. RNA sequencing data and clinical data of patients from HCC patients were obtained from The Cancer Genome Atlas (TCGA) and The International Cancer Genome Consortium (ICGC). Using data from TCGA as a training cohort (n=424) and data from ICGC as an independent external testing cohort (n=243), ERS-related genes were extracted to identify three common pathways IRE1, PEKR, and ATF6 using the GSEA database. Through univariate and multivariate Cox regression analysis, 5 gene signals in the training cohort were found to be related to ERS and closely correlated with the prognosis in patients of HCC. A novel 5-gene signature (including HDGF, EIF2S1, SRPRB, PPP2R5B and DDX11) was created and had power as a prognostic biomarker. The prognosis of patients with high-risk HCC was worse than that of patients with low-risk HCC. Multivariate Cox regression analysis confirmed that the signature was an independent prognostic biomarker for HCC. The results were further validated in an independent external testing cohort (ICGC). Also, GSEA indicated a series of significantly enriched oncological signatures and different metabolic processes that may enable a better understanding of the potential molecular mechanism mediating the progression of HCC. The 5-gene biomarker has a high potential for clinical applications in the risk stratification and overall survival prediction of HCC patients. In addition, the abnormal expression of these genes may be affected by copy number variation, methylation variation, and post-transcriptional regulation. Together, this study indicated that the genes may have potential as prognostic biomarkers in HCC and may provide new evidence supporting targeted therapies in HCC.

## Introduction

Hepatocellular carcinoma (HCC) is the fourth most common cause of cancer-related mortality worldwide  (1). It has been reported that most liver cancers are closely related to hepatitis B virus (HBV) infection and liver cirrhosis (2). Studies have shown that the morbidity and mortality associated with HCC continuing to increasing year on year and has become a major global public health problem (3). Clinical outcomes for HCC patients are difficult to predict based on classical histological classifications due to tumor heterogeneity (4). There is an urgent need to establish efficient and accurate risk assessment models to assess the prognosis in HCC patients to improve diagnosis and treatment.

Specific tumor biomarkers including α-fetoprotein, carcinoembryonic antigen, cytokines, nucleic acids and microRNAs have been used for early diagnosis and prognosis of HCC (5, 6). However, the prognosis remains unsatisfactory. Studies have revealed that ERS is positively associated with the occurrence and development of various human diseases including cancer (7), Studies have revealed that Pekinenin E can inhibit the growth of HCC by promoting ERS-mediated cell death and cell cycle arrest (8), and Endoplasmic reticulum stress can also promote HCC immune escape by transferring specific miRNAs to macrophages infiltrating the tumor microenvironment (9) yet the role of ERS-related prediction model in HCC remains to be determined.

In this study, we identified 374 cases of HCC that involved ERS-related genes from the TCGA database. A Cox regression analysis was applied to identify an ERS-related 5 gene signature that was used to construct a prognostic model. The model was validated using data from the ICGC database. We performed Kaplan-Meier survival and ROC (receiver operating characteristic) analyses to evaluate the performance of the model. The nomogram performed well in predicting the overall survival rate of HCC patients and can be used to predict prognosis in HCC. Based on comprehensive genomic data analysis, we aimed to demonstrate the value of an ERS-related signature to improve prognosis in HCC.

## Materials and Methods

### Acquisition of Gene Expression and Clinical Data

Harmonized RNA sequencing data (HTSeq-FPKM) and associated clinical information for Liver Hepatocellular Carcinoma (TCGA-LIHC) were obtained from TCGA database (https://portal.gdc.cancer.gov/). RNA-seq data included 424 cases, 374 of which were tumor samples and 50 adjacent normal tissues. The samples with survival time less than 90 days were deleted, and finally 329 samples with complete clinical information were used for analysis. The test dataset of gene expression and clinical trait data (the Liver Cancer-RIKEN JP) were downloaded from the ICGC database (https://dcc.icgc.org/). The ERS-related genes were obtained from the Molecular signatures Database (https://www.gsea-msigdb.org/gsea/msigdb/index.jsp), and involved the IRE1(M10426), PERK(M14369) and ATF6(M23457) pathways. Studies have shown that these three pathways play a pivotal role in the process of endoplasmic reticulum stress response (10). Then we obtained the expression levels of ERS-related genes in the training and testing cohort for subsequent analysis. Multi-omics used GSCAlite web tool (http://bioinfo.life.hust.edu.cn/web/GSCALite/) for subsequent analysis (11).

### Construction of ER Stress-Related Signals Related to Prognosis Signature

Univariate and multivariate regression analyses were conducted using the “survival together with forestplot” software package in R to screen ERS-related genes with significant prognostic value (p<0.01). Multivariate Cox regression analysis showed the minimum value of Akaike’s information criterion was 1032.78. This was the best cutoff point. The prognostic gene signature was constructed based on the linear combination of Cox regression coefficient multiplied by gene expression.

The risk score was calculated as = (coefficientGene _1_ × expression level of Gene _1_) + (coefficientGene _2_×expression level of Gene _2_) +… + (coefficient Gene _x_ × expression level of Gene _x_). According to the median risk score, the same parameters were used to classify patients in the training and validation cohorts into high and low-risk groups. We then used the “survival” package in R to draw a Kaplan–Meier survival curve to assess the survival rate between the high and low risk groups. The Receiver Operating Characteristic (ROC) curve and the area under the curve (AUC) over time were used to assess the predictive performance of the genetic and clinical factors in predicting survival at 1, 3 and 5 years. Also, the “pheatmap” package in R was used to illustrate the risk map. In the survival analysis, P-values < 0.05 were considered statistically significant and used for subsequent analysis

### External Verification of the Prognosis Gene Signature

We downloaded the corresponding liver cancer transcriptome data and clinical information from the ICGC database and used the same risk score formula to calculate the risk score for each patient. We adopted the median risk score as the best parameter to divide the patients into high and low risk groups. We then used the ROC and Kaplan-Meier curves to test the predictive performance of the prognostic gene model.

### Survival Analysis

Since the average survival time of HCC patients in clinical practice is about 6 months, in order to exclude the influence of premature death caused by postoperative infection, weakness, and other external factors, we filtered out samples with a survival period of less than 90 days. A total of 329 HCC patients were retained. The samples were divided into two groups based on each gene expression level, including the low and high expression groups. p value were calculated by the logrank test and the overall survival of patients was assessed by Kaplan-Meier survival analysis.

### Determination of Independent Prognostic Parameters

To determine the independent prognostic parameters and verify the independent prognostic value of gene signatures, we performed univariate and multivariate regression analysis including the 5-gene risk score and common clinic-pathological parameters (such as age, gender, stage, histological grade, vascular invasion, hepatitis virus infection and alcohol consumption). P-values of <0.05 were statistically significant.

### Construction of the Predictive Nomogram

Nomograms are widely applied to prognostic devices in oncology and medicine (12). The 5-gene signals determined by multivariate regression analysis were used as independent prognostic factors and included in the construction of the prognostic nomogram. The time-dependent receiver operating characteristic (ROC) curves were used to compare OS at 1, 3 and 5 years. We used the calibration chart and the consistency index (C index) to investigate the calibration and identification of the Nomogram (through the bootstrap method of 1000 resampling).

### Gene Set Enrichment Analysis (GSEA)

To clarify the underlying molecular mechanisms of the prognostic genetic models, Genomic Enrichment Analysis (GSEA) was used to explore the enrichment terminology in the pathway of the gene signature Kyoto Encyclopedia of Genes and Genomes (KEGG) related to ERS (13, 14). A p-value of < 0.05 was considered statistically significant.

### Comparison of the ERS-Related Gene Signature With Other HCC Prognostic Models

To determine whether our ERS-related 5-gene signature is superior to other HCC prognostic models, we used receiver operating curve (ROC) to compare 7-mRNA signature (15) and 9-mRNA signature (16). We obtained relevant genes in these models from the literature, and constructed 1 years ROC curves for the entire TCGA cohort. Then compared these gene prognostic models obtained from the literature with the ERS-related 5-gene signature of this study to evaluate the advantages and disadvantages of each.

### Multi-Omics Analysis of Five Gene Signatures

Multiple omics data are involved in this study regarding the selected target genes. We retrieved the whole exome sequencing results, copy number variants, methylation profile and miRNA expression profile from the TCGA GDC portal. The maf format file including the genomic variants was downloaded by maftools R package. The other omics data were retrieved by TCGAbiolinks R package. All the transcripts, mutations, probes and miRNAs are restricted to the target genes.

### Statistical Analysis

The R software package (4.0.2) was used for statistical analysis. A Pearson χ2 test and Fisher’s exact test were used to analyze the qualitative variables.

## Results

### Identification of Endoplasmic Reticulum Stress Related Genes

First, we identified three gene sets associated with ERS-related by searching the keyword “IRE1 PERK ATF6” in the MSigDB database. To investigate the expression level of these genes in HCC, we downloaded RNA-seq data from TCGA. In 329 liver cancer specimens, 88 genes of ERS-related were identified, The expression profiles of the 88 ERS- related genes in the training cohort were used to establish a prognostic model. The clinical-pathological characteristics of liver cancer patients from the two databases were shown in **Table 1**.

**Table 1:** The clinical characteristics of liver cancer patients in the TCGA and ICGC cohorts.

Clinical characteristics		Total	%
**TCGA**		329	100
Survival status	Alive	221	67.17
	Death	108	32.83
Age	≤65	210	63.83
	>65	119	36.17
Gender	Female	103	31.31
	male	226	68.69
Grade	G1	50	15.2
	G2	154	46.81
	G3	107	32.52
	G4	13	3.95
	unkonw	5	1.52
Stage	I	155	47.11
	II	74	22.49
	III	76	23.1
	IV	3	0.91
	unkonw	21	6.39
T classification	T1	162	49.24
	T2	81	24.62
	T3	71	21.58
	T4	13	3.95
	unkonw	2	0.61
Race	asian	146	44.38
	white	159	48.33
	unkonw	24	7.29
Child-Pugh score	A	198	60.18
	B	20	6.08
	C	1	0.3
	unkonw	110	33.44
Histologic grade	G1	50	15.2
	G2	155	47.11
	G3	107	32.52
	G4	12	3.65
	unkonw	5	1.52
**ICGC**		228	100
Survival status	Alive	189	82.89
	Death	39	17.11
Age	≤65	89	39.04
	>65	139	60.96
Gender	Female	61	26.75
	male	167	73.25
Stage	I	36	15.79
	II	106	46.49
	III	67	29.39
	IV	19	8.33
Prior_malignancy	NO	198	86.84
	YES	30	13.16

### Construction and ICGC Verification of ERS-Related Gene Signals

**Figure 1:**
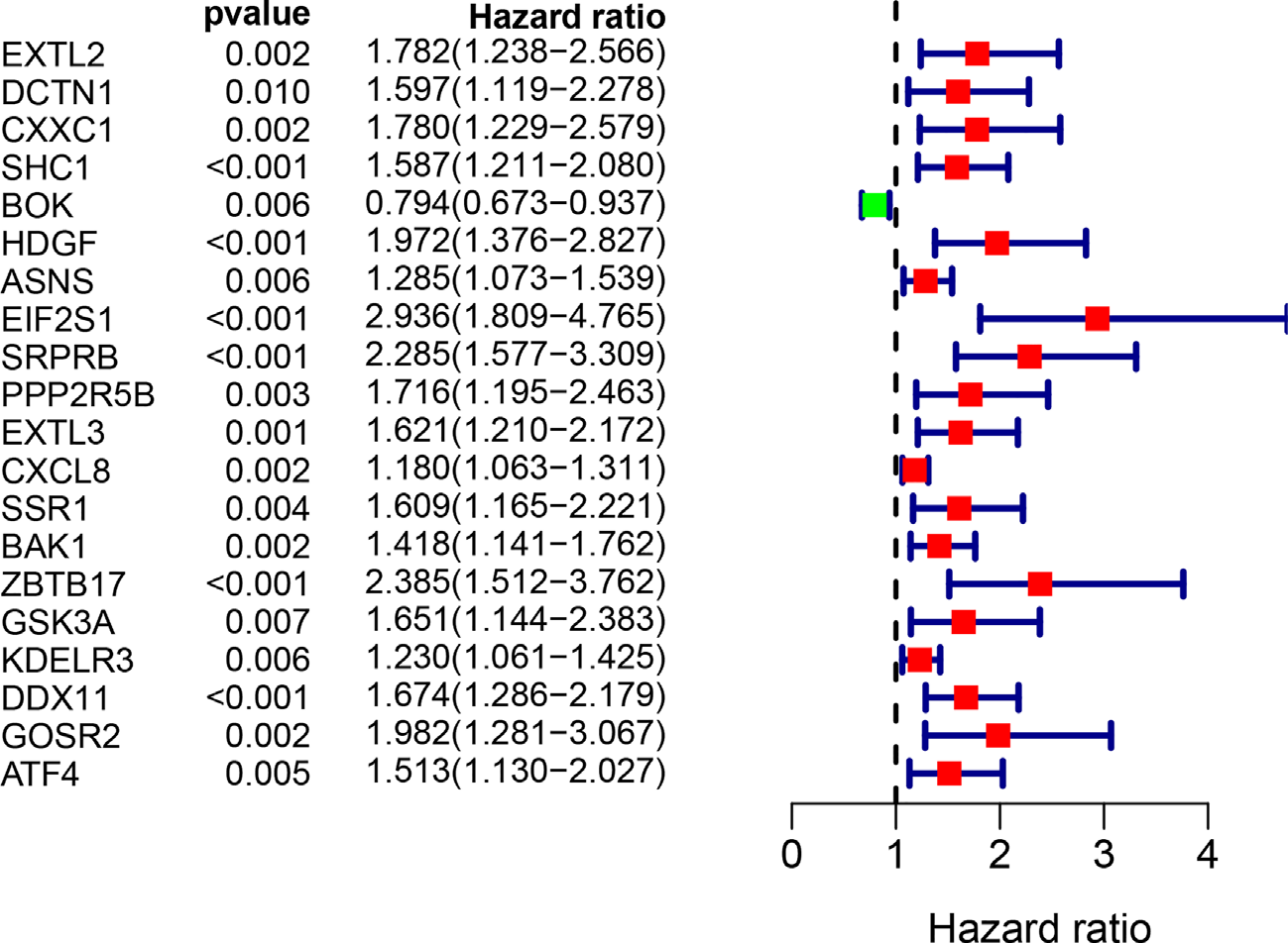
Univariate Cox regression analysis of 20 genes related to ERS in the training set of TCGA cohort.

The univariate Cox regression model was used to identify a total of 20 ERS-related genes related to overall survival (OS) (**Figure 1**). Further multivariate Cox regression analysis was performed and finally five ERS-related genes were defined and used to create a prognostic model. Multivariate Cox regression analysis of the five ERS-related genes in the training cohort is shown in **Table 2**.

**Table 2:** Multivariate Cox regression analysis of the 5 genes associated with ERS in the training set of TCGA cohort.

Id	Coef	HR	HR.95L	HR.95H	P value
HDGF	0.305209	1.356909	0.910073	2.023135	0.134238
EIF2S1	0.768559	2.156657	1.35023	3.444723	0.001297
SRPRB	0.650264	1.916047	1.26283	2.907151	0.002236
PPP2R5B	0.501056	1.650464	1.139218	2.391142	0.008071
DDX11	0.294516	1.342476	0.986678	1.826576	0.060848

**Figure 2:**
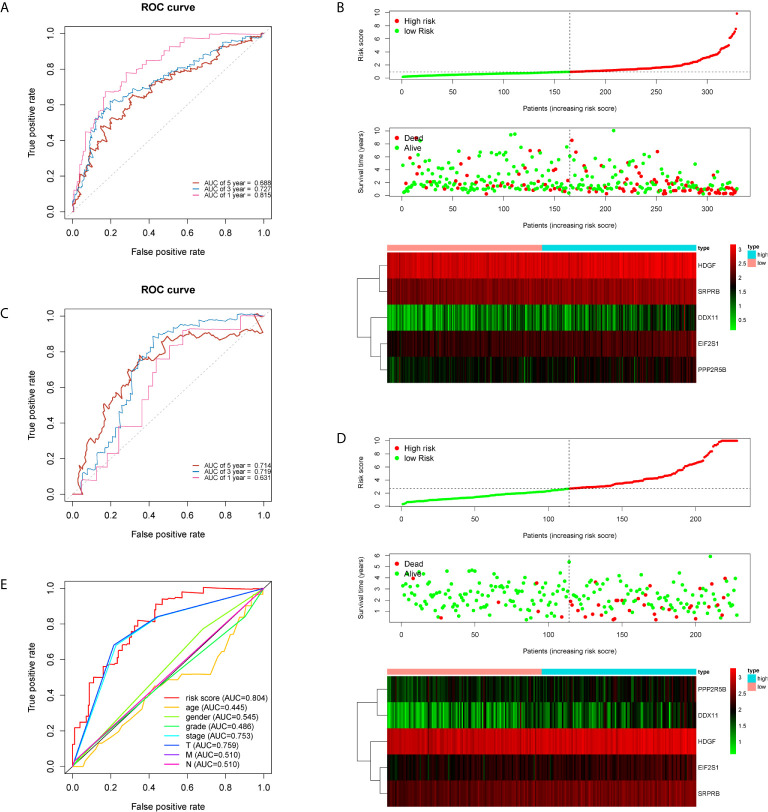
Time-dependent ROC analysis and risk scoring analysis were used for the 5-gene signature in HCC. Time-related ROC analysis **(A)**, and risk score **(B)** in the training set of TCGA cohort. Time-related ROC analysis **(C)**, and risk score **(D)** in the testing set of ICGC cohort. multi-index ROC analysis in the TCGA cohort **(E)**.

The five genes related to ERS include *HDGF, EIF2S1, SRPRB, PPP2R5B and DDX11*. The model risk score =0.305* HDGF+0.769* EIF2S1+0.650* SRPRB+0.501* PPP2R5B+0.295* DDX11. Taking the median risk score (0.929 for the training cohort and 2.671 for the test cohort) as the threshold, the patients were separated into high and low-risk groups. Time-dependent ROC (AUC value) was applied to evaluate the prognostic ability of the 5-gene signals. In the training cohort, the AUC values of 1, 3 and 5 years OS predictions for the risk score were 0.815, 0.727 and 0.688 respectively (**Figure 2A**). However, in the test cohort, the AUC values of 1, 3 and 5 years were 0.631, 0.719 and 0.714 (**Figure 2C**). With increasing risk scores, the mortality rate of patients in the training and test cohorts gradually increased (**Figures 2B, D**). Subsequently, we also performed ROC analysis to evaluate whether 5-gene risk score can predict a better prognosis compared with common clinicopathological characteristics. As shown in the **Figure 2E**, the 5-gene risk score (AUC=0.804) has a better predictive effect on the prognosis of HCC than age, gender, stage, grade and TNM stage. These results indicate that the prognostic model has good predictive power and specificity.

### Relationships With Clinicopathological Characteristics and Survival Prognosis

**Figure 3:**
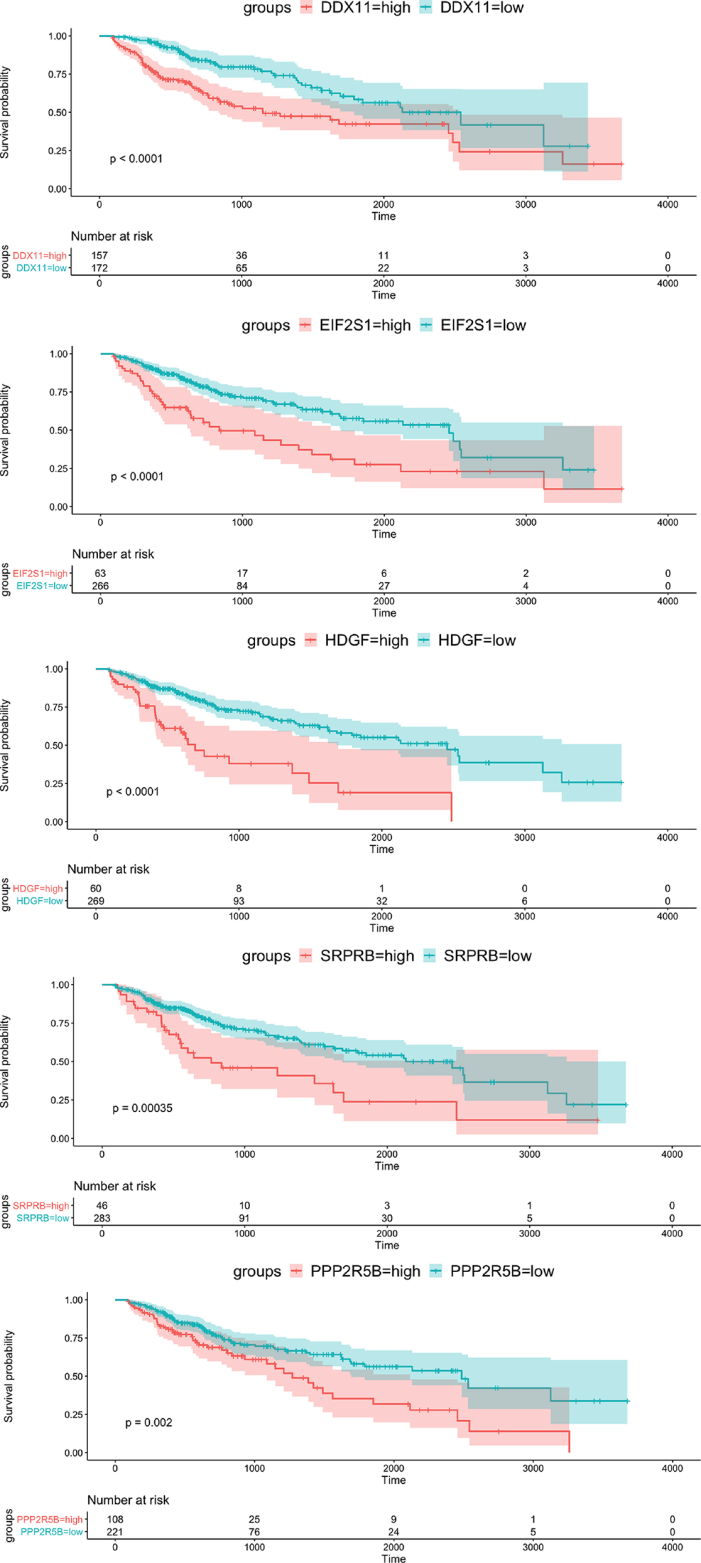
Kaplan-Meier survival curve of OS for 5 hub genes with survival significance in the TCGA cohort. Red and green colors represent high and low expression, respectively.

To verify the prognostic value of the 5 genes, we divided the samples into high expression group and low expression group according to the expression value of each gene. The Kaplan-Meier survival analysis showed that patients with high expression of the five target genes tend to have a poor prognosis, while the low expression samples have a relatively good prognosis (**Figure 3**).

**Figure 4:**
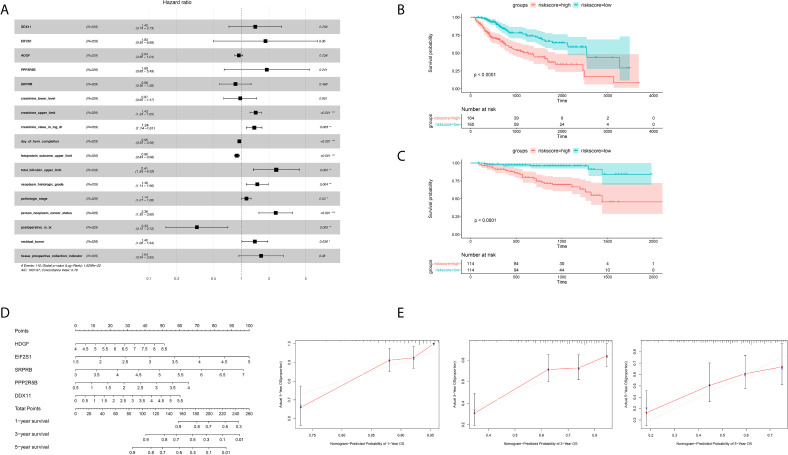
Identification of independent prognostic factors and establish forecast nomogram. **(A)** Forrest chart of HCC multivariate cox analysis in the TCGA cohort. **(B)** Kaplan-Meier curve of 5 gene markers in the TCGA population for the training set. **(C)** Kaplan-Meier curve of 5 gene markers in the ICGC population for the testing cohort. **(D)** Establishment of nomogram combining 5 gene markers in the TCGA cohort. **(E)** The Calibration curves, used for internal verification nomogram in the TCGA cohort. *p < 0.05, **p < 0.01, ***p < 0.001.

In order to further integrate clinical information to achieve multivariate survival analysis, we collected all clinical information records of liver cancer patients in the TCGA database, and filtered out indicators with missing values of more than 50%, and finally retained 35 clinical indicators, including age, gender, staging, Classification. We used the multivariate cox regression algorithm to identify clinical features that significantly affect the prognosis. For the results of the multivariate cox regression analysis, see the **Supplementary Table S1**. To better visualize the influence pattern of each clinical indicator on the prognosis, we used the significant clinical features to construct a forest map model, as shown in **Figure 4A**. It can be seen that whether to receive chemotherapy and radiotherapy after surgery were protective factors for survival and prognosis. Conversely, factors such as tumor status, tumor residual, creatinine value, total bilirubin value, histologic grade and pathological stage are risk factors. Next, to determine whether the prognostic model is independent of the common clinical features that predict the prognosis of HCC, we used two independent TCGA and ICGC data sets to perform univariate and multivariate Cox regression analysis. The results obtained consistently indicate that the prognostic risk model constructed based on endoplasmic reticulum stress-related genes is a significantly independent factor (**Table 3**). After integrating all 5 target genes and clinical information, the predictive performance of the risk model for TCGA samples and the predictive performance on independent verification data were shown in (**Figures 4B, C**). Results analysis revealed that the multivariate risk model can significantly distinguish two patients with different survival prognosis on both TCGA samples and independent validation data, and the logrank test p value is less than 0.0001. The nomogram of 1, 3 and 5 years OS in the cohort were shown in **Figure 4D**. The calibration chart shows the accuracy of assessing the prediction nomogram. The nomogram performed well when predicting the probability of survival at 1, 3 and 5 years (**Figure 4E**).

**Table 3:** Univariate and multivariate Cox regression analysis of prognostic factors of hepatocellular carcinoma patients in TCGA and ICGC cohorts.

Variables	Univariate analysis		Multivariate analysis	
	HR (95%C I)	P	HR (95%CI)	P
**TCGA**				
Risk_score(high- vs. low-risk)	2.248(1.366-3.699)	0.001	2.044(1.232-3.393)	0.006
Age(≥65 vs. <60 years)	1.217(0.747-1.980)	0.43		
Gender(male vs. female)	0.767(0.468-1.259)	0.294		
Histologic_grade (3-4 vs. 1-2)	1.270(0.784-2.056)	0.331		
Stage(3-4 vs.1-2)	2.611(1.595-4.274)	<0.001	2.290(1.345−3.898)	0.002
Vascular_invasion (yes vs. no)	1.350(0.818-2.227)	0.24		
Hepatitis_virus_infection (yes vs. no)	0.755(0.449-1.270)	0.29		
Alcohol_consumption (yes vs. no)	0.696(0.378-1.279)	0.243		
**ICGC**				
Risk_score(high- vs.low-risk)	6.679(2.791-15.984)	<0.001	6.099(2.542-14.631)	<0.001
Age(≥65 vs. <60 years)	1.056(0.552-2.020)	0.87		
Gender(male vs.female)	0.433(0.228-0.824)	0.011	0.378(0.193-0.740)	0.005
Stage(3-4 vs.1-2)	2.016(1.074-3.784)	0.029	2.139(1.104-4.145)	0.024

CI, confidence interval; HR, hazard radio.

### Principal Component Analysis (PCA)

**Figure 5:**
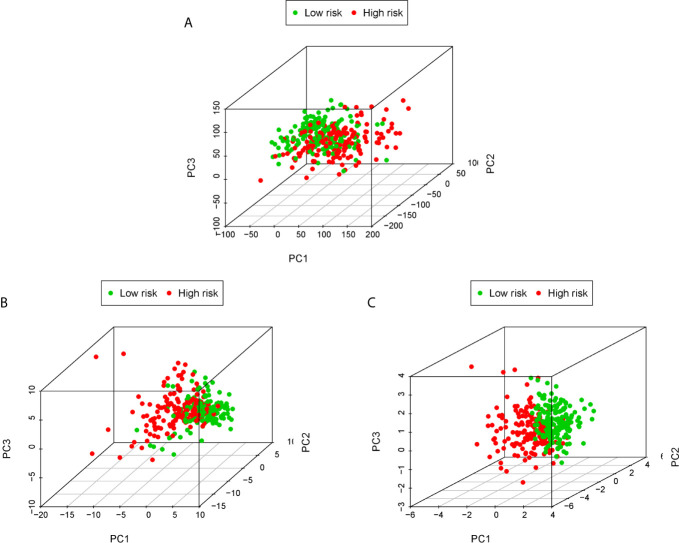
PCA analysis of each genome in the TCGA cohort. PCA analysis based on the entire genome expression profile **(A)**, the all genes related to ERS **(B)** and risk model genome **(C)**.

PCA is often used to visualize the distribution of risk amongst different populations in line with the risk gene sets, ERS gene sets and the whole genome expression sets. We performed PCA analysis on the entire gene expression profiles and showed unclear separation in risk statuses (**Figure 5A**). According to the all genes related to ERS, the low and high-risk groups tended to be divided into two aspects. Patients in the low and the high-risk groups had different ERS status (**Figure 5B**). However, when based on the risk model genome there were significant differences between the high and low risk groups, shown a clearly separated state (**Figure 5C**).

### Gene Set Enrichment Analysis (GSEA)

**Figure 6:**
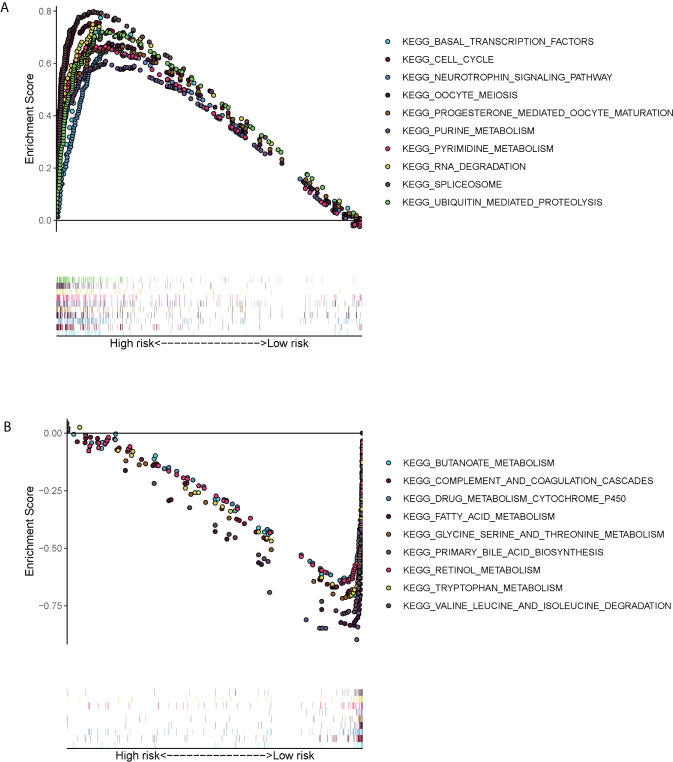
Gene set enrichment analysis (KEGG pathways) in the TCGA cohort. **(A)** Enrichment pathways for high-risk patients. **(B)** Enrichment pathways for low-risk patients.

GSEA showed that the ERS-related gene signature was commonly concentrated in the basal transcription factors, cell cycle neurotrophin signaling pathway, oocyte meiosis, progesterone mediated oocyte maturation, purine metabolism, pyrimidine metabolism, RNA degradation, spliceosome and ubiquitin mediated proteolysis in high-risk patients (**Figure 6A**). In low-risk patients the main pathways involved were butanoate metabolism, complement and coagulation cascades, drug metabolism cytochrome p450, acid metabolism, glycine serine and threonine metabolism, primary bile acid biosynthesis, retinol metabolism, tryptophan metabolism, valine leucine and isoleucine degradation (**Figure 6B**).

### Comparison of the ERS-Related Gene Signature With Other HCC Prognostic Models

**Figure 7:**
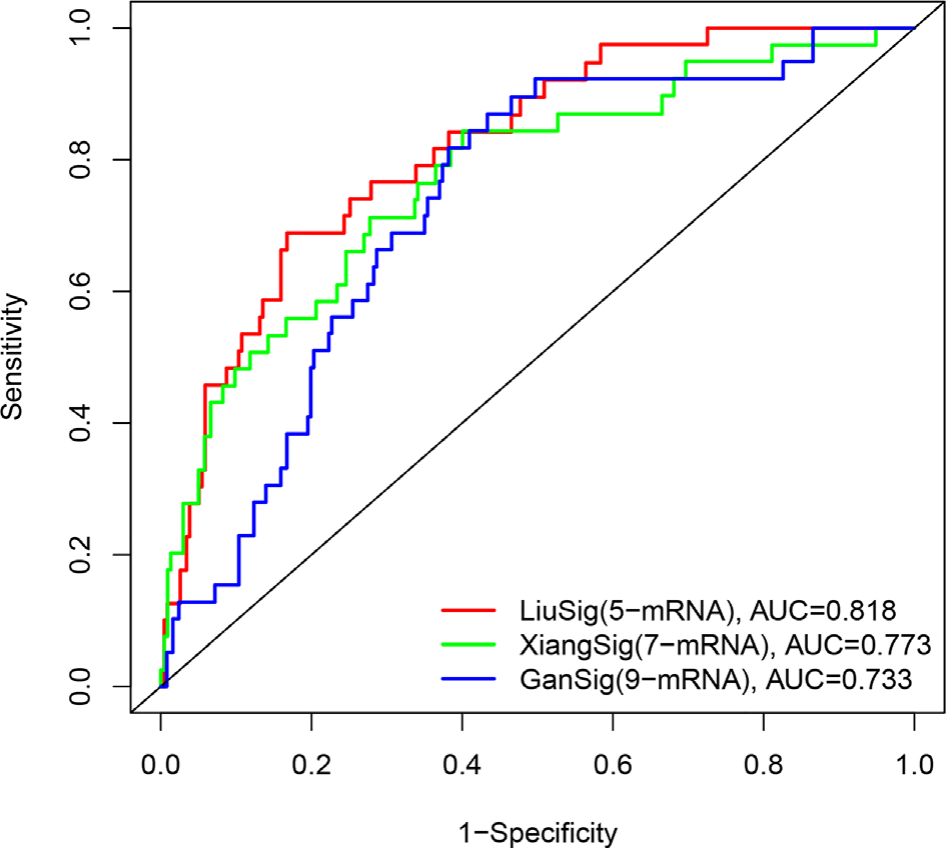
The ROC analysis at 1 years of overall survival for our ERS-5 gene, Xiang-7 gene and Gan-9 gene signatures in the entire TCGA cohort.

To determine whether our ERS-related 5-gene signature is superior to other HCC prognostic models, we compared 7-mRNA signature (15) and 9-mRNA signature (16) for the entire TCGA cohort. Among the two prognostic models, 7-mRNA signature show that its 1 year AUC values was 0.773, the 9-mRNA signature show that its 1 year AUC values was 0.733. The above results indicate that two models have certain predictive power. However, compared with our 5-gene signature of this study, the predictive power of the ERS-related 5-gene signature is significantly higher than the three prognostic models (**Figure 7**).

### Multi-Omics Analysis of Five Gene Signatures

Through above systematic statistical analysis, we found that the expression levels of 5 target genes can significantly interfere with the survival and prognosis of liver cancer patients. In order to further investigate the biological mechanism of the abnormal expression of the five target genes, we analyzed the five genes from different omics dimensions, including the genome level, copy number level, methylation level and post-transcriptional regulation level.

**Figure 8:**
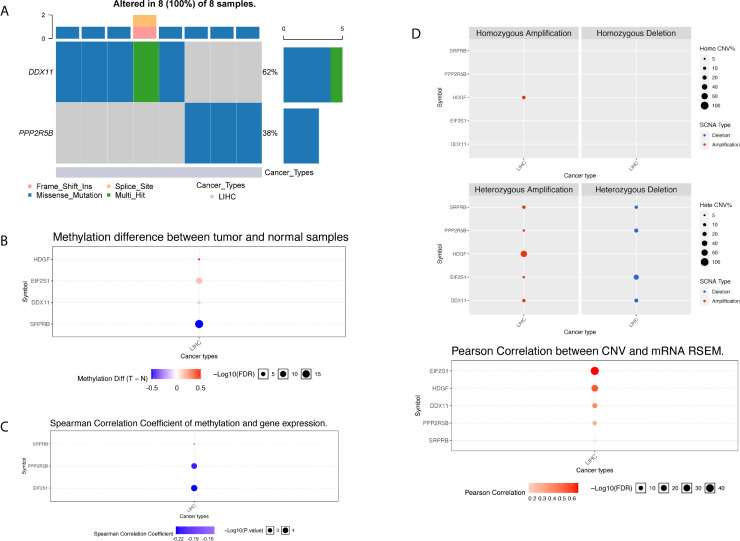
Multi-omics analysis of five gene signatures in TCGA cohort by GSCALite. **(A)** In the oncoplot graph, different color represents different types of variation. **(B)** Methylation difference between tumor and normal samples. **(C)** Spearman correlation coefficient of methylation and gene expression. **(D)** Copy number variation pattern of 5 gene signatures.

We used the R package maftools to obtain all exon variation data of liver cancer samples in maf format. The mutation data contains all liver cancer somatic mutations identified by the MuTect2 algorithm. Two of the five target genes have high frequency mutations in liver cancer patients, and they were mainly missense mutations. As shown in **Figure 8A**, the gene DDX11 has mutations in 5 liver cancer samples, while PPP2R5B has mutations in 3 samples. Since the five target genes showed differential expression in most liver cancer samples, this result suggests that the abnormal expression of the five target genes may not be related to mutations.

We further investigated the abnormality of methylation level. There were four genes at the methylation level that were significantly different in tumor patients and normal tissues, as shown in **Figure 8B**. The gene HDGF and EIF2S1 were mainly hypermethylation, while the DDX11 and SRPRB were hypomethylation, the most significant of which were SRPRB and EIF2S1. As we all know, abnormal methylation plays an important role in the regulation of expression level, so we further compared the correlation between methylation level and expression level. It was found that there was a correlation between the differences in methylation and expression levels of three genes, and the results are shown in **Figure 8C**. Hypermethylation tends to inhibit gene expression, so the significant negative correlation suggests that the cause of expression differences may be caused by abnormal regulation of the apparent level. Although abnormal expression levels may be affected by multiple dimensions of omics, the correlation between methylation and expression levels suggests that the expression levels of SPPRB, PPP2R5B and EIF2S1 were regulated by abnormal methylation.

Next, we compared the copy number variation from two perspectives of heterozygosity and homozygosity, and found that the main manifestations of liver cancer patients were loss of heterozygosity and amplification. The HDGF gene was all amplified variants, as shown in **Figure 8D**. At the same time, we found that there was a significant correlation between the copy number variation and the expression level variation of the 5 genes, suggested that the genome copy number variation affects the quantification of gene expression in RNAseq. Therefore, abnormal gene expression may be due to the combined effect of copy number variation and methylation variation.

### Establishment of Mirna-mrna Regulatory Network

**Figure 9:**
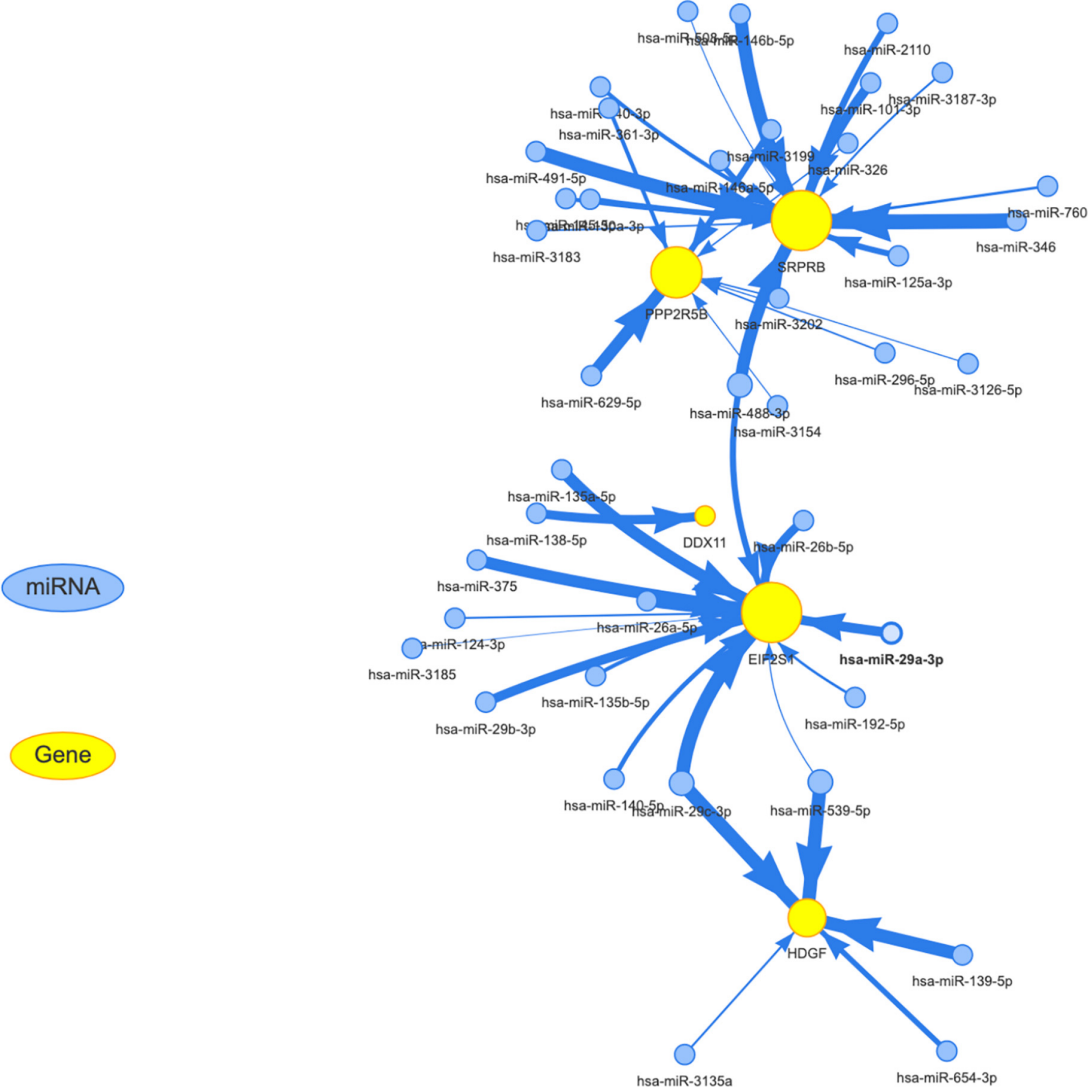
Establishment of miRNA-mRNA regulatory network. The yellow and blue circles represent the target gene and miRNA, respectively. The arrows represent regulatory relationships.

We know that in addition to the effects of mutation, methylation, and copy number on expression levels, post-transcriptional regulation also plays an important role. The most important noncoding rna is mirna. Through the analysis of the mirna target database, we obtained mirna for targeted regulation of 5 target genes. for the specific results, see the **Supplementary Table S2**. The regulatory relationship between mirna and target genes was shown in **Figure 9**. The yellow and blue circles in the figure represent the target gene and mirna, respectively. The arrows represent regulatory relationships. We found that some mirna can simultaneously regulate multiple target genes. Since target genes are significantly related to the prognosis of liver cancer, if the mirna involved in post-transcriptional regulation is also significantly related to the prognosis, it suggests that the regulatory relationship of mirna-gene plays an important role in the progression of liver cancer.

**Figure 10:**
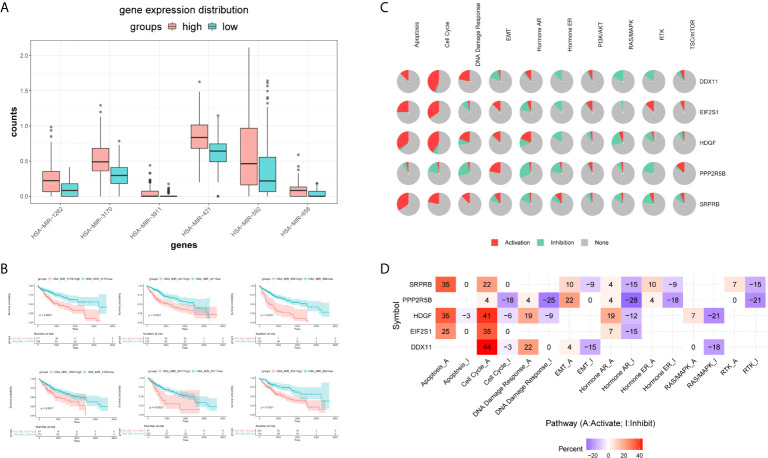
Expression distribution and survival analysis of miRNA in the TCGA dataset. **(A)** miRNA expression distribution in different risk groups. **(B)** KM survival analysis of 6 miRNAs. **(C)** Functional analysis of 5 target genes. Red and green in the pie chart represent the direction and proportion of expression. **(D)** Inferred activity of the identified 5 target genes in biological pathways. A and I to mark the active and inhibited pathways, respectively.

Firstly, we used the mirna expression data of liver cancer samples downloaded from the TCGA database. Then, all miRNAs were screened by Univariate Cox regression, and the most significant top 6 miRNAs were screened according to P value less than 0.01, and the results were shown in the **Supplementary Table S3**. Subsequently, boxplot was used to visualize the expression distribution of 6 miRNAs in patients with different risk of HCC, as shown in **Figure 10A**. The expression level of mirna is significantly different in different risk groups. We further used the KM curve to analyze the relationship between the expression of 6 mirna and the survival prognosis of patients, as shown in **Figure 10B**. All the 6 miRNAs were significantly correlated with the prognosis of HCC patients. The main target genes involved in the regulation of these six miRNAs were EIF2S1 and HDGF. Therefore, the post-transcriptional regulation of EIF2S1 and HDGF plays a key role in the progression of liver cancer patients.

Pathway analysis showed that the five target genes were mainly involved in regulating apoptosis, cell cycle, hormone receptors and other cancer-related pathways (**Figure 10C**). In order to further study whether each pathway was activated or inhibited, we used A and I to mark the active pathway and the inhibited pathway, as shown in **Figure 10D**. It was obvious that the apoptosis pathway was activated in liver cancer patients under the regulation of five genes. The RAS/MAPK and RTK pathways were mainly inhibited. Cell cycle, DNA damage response, hormone receptors, and EMT pathways were both activated and/or inhibited.

## Discussion

The complexity of tumor heterogeneity in HCC remains a major challenge to accurate patient prognosis. It is particularly important to search for new prognostic biomarkers and to establish more accurate prognostic models to predict the survival of liver cancer patients. Many previous studies have focused on mRNA gene signatures and achieved promising results in predicting the prognosis of HCC (16–19). However, the predictive abilities of these biomarkers are limited and there is a need to develop more accurate signatures. Experimental data suggest that ER stress and unfolded protein response are involved in HCC development, aggressiveness and response to treatment, However, the exact role of ERS stress in HCC needs further study (20). In this study, we aimed to discover a novel prognostic marker related to ERS to provide new approaches in the treatment of liver cancer.

In this study, we created a new 5-gene signature based on ERS (including *HDGF, EIF2S1, SRPRB, PPP2R5B and DDX11*) for prognosis in HCC. Our 5-gene signature was demonstrated as an independent prognostic factor in HCC and the overall survival of the high-risk group was significantly lower than the low-risk group. The nomogram combining the 5-gene markers showed that it performed best in predicting survival indicating that the risk model and had utility in evaluating the prognosis of HCC. GSEA analysis revealed a series of significantly enriched oncology features and some metabolic processes in the two groups that may help to understand the underlying molecular mechanism of the disease.

HDGF is a critical regulator of cancer cell activities and plays central roles in transformation, apoptosis, angiogenesis and metastasis in several cancer types (21–24). In HCC, HDGF is considered a liver cancer-derived growth factor and it has been shown that targeted inhibition of HDGF may have efficacy as a new type of HCC treatment (25). EIF2S1 is a phosphorylated form of eIF2α that inhibits eIF2α-mediated translation Studies have shown that it is related to oxidative stress and ER protein folding and acts to maintain the differentiation state of β-cells (26). However, the role and mechanism of EIF2S1 in HCC remain unclear.

SRPRB is a novel human gene whose transcript is upregulated in apoptotic MCF-7 cells (27). It is commonly expressed in the liver, pancreas, thyroid, testis and 25 other tissues in the human body. Studies have shown that SRPRB regulates HHCC cell proliferation and apoptosis by down-regulating TIMP3 and up-regulating CDK inhibitor p21. Also, it may interact with SERP1 to affect the prognosis of pancreatic cancer (28, 29).

The mechanism of SRPRB in the prognosis of liver cancer merits further investigation. PPP2R5B is the regulatory subunit of protein phosphatase 2 (PP2A). As PP2A is generally recognized as a tumor suppressor, studies have shown that highly expressed mitotic regulators and low-expressed PP2A class I subunits (such as PPP2R5B) can improve survival rate (30). Previous studies have also shown that SRPRB is responsible for the dephosphorylation and inactivation of Akt protein and can lead to insulin resistance in adipocytes induced by chronic hyperinsulinemia (31).

The role of PPP2R5B in liver cancer remains unclear. DDX11 is a DNA helicase, involved in the cohesion of sister chromatids and DNA repair pathways (32). The expression of DDX11 is dysregulated and has a significant effect on the progression of many tumors such as lung adenocarcinoma (33), melanoma (34) and Warsaw breakage Syndrome (35). Some evidence has shown that DDX11 regulates the proliferation and invasion of HCC cells by activating the PI3K/AKT/mTOR signaling pathway (36).

Hepatocellular carcinoma is a multistep process of genetic and epigenetic alterations involving many genes. Recent studies have shown that multi-omics data integration strategies spanning different cell function levels across the genome, epigenome, transcriptome, proteome, metabolome, and microbiome provide a new perspective for the typing, diagnosis and prognosis of complex diseases (such as cancer) (37). With the advancement of high-throughput technology, the mutation, methylation, copy number and gene expression patterns of various cancer types have been identified and characterized. Previous reports in the literature usually indicate that mutations in driving oncogenes are associated with poor prognosis. For example, the PIK3CA mutation in breast cancer (38), the NBN mutation in prostate cancer (39), and the TP53 mutation in gastric cancer (40). However, the specificity and sensitivity of this statement needs further confirmation. As we all know, copy number variation (CNV) is generally considered the source of genetic variation, and its importance has been confirmed in recent studies (41). In addition to gene mutations, CNV also plays a very important role in the occurrence and development of many cancers, such as ovarian cancer (42), bladder cancer (43), gastric cancer (44), and so on. A recent study analyzed the genome profiles of 17,879 tumors in patients with known outcomes, and found that almost all mutations in cancer driver genes contained very little information about the patient’s prognosis. However, the copy number changes in these same driver genes have obvious prognostic power (45). It indicates that the identification of copy number may be an important target for future tumor treatment. As an important epigenetic modification, DNA methylation participates in the regulation of gene transcription and maintains the stability of the genome (46). Methylation changes, usually including hypomethylation of proto-oncogenes and methylation of tumor suppressor genes, play a key role in regulating the expression of many tumor genes. In this study, we found that the abnormal expression of 5 genes may be due to the combined effect of copy number variation and methylation variation, and its abnormal expression may not be related to mutation. Multi-omics analysis of these genes will deepen the understanding of the occurrence, development and potential mechanism of liver cancer.

A 5-gene model and the nomograms related to stresses are yet to be rarely reported. Our biomarker may be a useful tool in the prognosis and treatment of HCC. However, our study had several shortcomings including the need for a lack of external validation. In addition, due to the limitations of the database, the clinical data of some important parameters were incomplete, such as BCLC staging, preoperative AFP, and CEA were excluded from the scoring system analysis. It may affect statistical power. Therefore, our study cannot rule out that the survival of patients may be affected by other key clinical factors. Although the 5-gene model had implications for the diagnosis and treatment of liver cancer, more complex mechanisms associated with the prognosis of liver cancer require further exploration.

## Conclusions

Our study indicated that a 5-gene signature based on ERS-related independent prognostic significance could potentially serve as a prognostic indicator for clinical decision-making in the treatment of HCC patients. Multi-omics analysis of the role of these genes in promoting tumors in liver cancer will provide a new perspective for elucidating the potential mechanisms of liver cancer prognosis.

## Data Availability Statement

The datasets presented in this study can be found in online repositories. The names of the repository/repositories and accession number(s) can be found in the article/**Supplementary Material**.

## Author Contributions

PL conceived and designed this study. The software and the data analyses were carried out by PL and JW. Other authors including FM, ZX, HD and YD also participated in the data analyses of this study. The manuscript was written by PL and supervised by JW. All authors read and approved the manuscript and agree to be accountable for all aspects of the research in ensuring that the accuracy or integrity of any part of the work was appropriately investigated and resolved. PL and JW have contributed equally to this work. All authors contributed to the article and approved the submitted version.

## Funding

This work was supported by a grant from the National Natural Science Foundation of China (No. 81772516).

## Conflict of Interest

The authors declare that the research was conducted in the absence of any commercial or financial relationships that could be construed as a potential conflict of interest.
